# Bilingual and Monolingual Idiom Processing Is Cut from the Same Cloth: The Role of the L1 in Literal and Figurative Meaning Activation

**DOI:** 10.3389/fpsyg.2016.01350

**Published:** 2016-09-09

**Authors:** Sara D. Beck, Andrea Weber

**Affiliations:** ^1^SFB 833, University of TübingenTübingen, Germany; ^2^Psycholinguistics and Applied Language Studies, English Department and SFB 833, University of TübingenTübingen, Germany

**Keywords:** cross-modal priming, L2 listening, figurative language, idioms, translation

## Abstract

The present study examines non-native (L2) and native (L1) listeners' access to figurative idiomatic meaning and literal constituent meaning in two cross-modal priming experiments. Proficient German learners of English (L2) and native speakers of American English (L1) responded to English target words preceded by English idioms embedded in non-biasing prime sentences in a lexical decision task. English idioms differed in levels of translatability: *Lexical level idioms* had word-for-word translation equivalents in German, while *post-lexical level idioms* had matching idiomatic concepts in German but could not be translated word for word. Target words either related to the figurative meaning of the idiom or related to the literal meaning of the final constituent word of the idiom (e.g., *to pull someone's leg*, literal target: *walk*, figurative target: *joke*). Both L1 and L2 listeners showed facilitatory priming for literally- and figuratively-related target words compared to unrelated control target words, with only marginal differences between the listener groups. No effect of translatability was found; that is, the existence of word-for-word translation equivalents in German neither facilitated nor hindered meaning activation for German L2 listeners. The results are interpreted in the context of L1 and L2 models of idiom processing as well as further relevant translation studies.

## Introduction

While understanding idioms is usually easy for native (L1) listeners of a language, non-native (L2) listeners often find recognizing and understanding them to be a stumbling block. Like other facets of figurative language, idioms are both complex and pervasive in language; e.g., Pollio, Barlow, Fine, and Pollio (as cited in Cooper, 1999) estimated an average of 7000 idioms a week for L1 speakers based on the occurrence of figurative language analyses of political debates, psychology texts, novels, and psychotherapy sessions. Though idioms are not a homogenous group, researchers can generally agree that idioms are multi-word expressions with limited variation in syntactic structure. Additionally, the meaning of an idiom typically differs from the literal meanings of the individual constituent words (see e.g., Liu, 2008). The fixed nature of idioms might suggest that non-native speakers can learn and use idioms quickly, but the conventional misalignment between figurative and literal meaning poses a particular challenge. For example, when a speaker expresses that she is *in hot water*, one can interpret from the figurative meaning that she is in trouble rather than assuming that she is literally submersed in heated water, as in a hot bath or hot springs. What might be obvious to a native listener might not be to a non-native listener, and the expression could confuse rather than inform an L2 listener about the situation. L2 proficiency would, however, benefit significantly from mastery of L2 idiomatic expressions: not only would it make L2 speakers sound more native-like (Boers et al., 2006) it would also free up processing capacities since fixed multi-word expressions are known to be easier to process than novel phrases (Pawley and Syder, 1983; Conklin and Schmitt, 2008; Siyanova-Chanturia, 2013). In fact, an ability to produce and comprehend idiomatic expressions is one of the measurements used to determine the English level of non-native users (see e.g., Common European framework of reference for languages, 2001). Compared to the broad research on L1 idiom processing, a number of questions remain for the L2. In the studies presented here, we addressed two major questions: How does L2 access to figurative meaning of an idiom and literal meaning of constituent words compare to L1 access? And, what is the role of the L1 in this process? More specifically, can L1 similarities, i.e., translatability, ease L2 processing? To address these questions, we tested online processing of figurative and literal meaning in idioms in L1 and L2 listeners. Additionally, we explored the impact of the L1 on the L2 by addressing the translatability of the idioms from the L2 into the L1 and tested idioms that were either directly translatable or not translatable.

L1 idiom processing research, though diverse in focus, has put weight on the comparisons between processing literal and figurative meaning as well as processing idioms and novel phrases. The fact that idioms often have both a literal and a figurative interpretation has provided for a rich field of research (Cacciari and Tabossi, 1988; Libben and Titone, 2008; Tabossi et al., 2009; Cacciari and Corradini, 2015). While the figurative meaning of an idiom is conventionalized and known to native speakers, its literal interpretation can be either logical, nonsensical, or somewhere in between. Though it is possible that someone is bathing in the example of being *in hot water* (with an idiomatic or figurative interpretation denoting “in trouble”), in the idiom *to be on cloud 9* (with a figurative interpretation of “being very happy”) there is not a likely, logical interpretation in the real world in which a person can be found on a cloud called “9.” Furthermore, when considering the literal interpretation of an idiom, research can remain on the phrasal level or can consider access to the literal meaning of the constituent parts. When again considering the idiom *in hot water*, we can focus on access to the figurative interpretation, “in trouble,” access to the whole interpretation of the literal phrase, “to be in heated water such as a bath or hot springs,” or we can consider access to the meanings of the individual constituent words such as “hot” or “water.”

In the presence of such diverse processing possibilities, various theoretical approaches to the processing of idioms concerning access to literal constituent meaning and figurative meaning have been put forward in the last decades. Early approaches treated idioms homogenously and suggested two individual modes of processing for literal and figurative meaning. These approaches suggested that processing occurred in stages which had, generally, three possibilities: literal meaning first (e.g., Bobrow and Bell, 1973), figurative meaning first (e.g., Gibbs, 1980), and simultaneous processing (e.g., Swinney and Cutler, 1979). These stage approaches to idiomatic processing often assumed figurative meaning as part of retrieval, while literal processing involves composition. Evidence from psycholinguistic studies quickly showed that, in some cases, figurative meaning is available more quickly than literal meaning (Ortony et al., 1978; Swinney and Cutler, 1979; Cacciari and Tabossi, 1988), discounting purely literal-first processing approaches and pushing for simultaneous processing models or more complex models. Furthermore, such models of processing have been criticized as idioms have been more systematically categorized and labeled based on their diverse properties (Gibbs et al., 1989; Nunberg et al., 1994; Titone and Connine, 1994b) such as literality (Titone and Connine, 1994b), predictability (Cacciari and Tabossi, 1988), decomposability (Gibbs et al., 1989), familiarity (Tabossi et al., 2009), saliency (Giora, 1997), and more recently emotional valence and arousal (Citron et al., 2016). Theoretical approaches and experimental methods have adapted to incorporate psycholinguistic findings on such idiomatic properties. For instance, both familiarity (see e.g., Libben and Titone, 2008 for an overview) and a strongly biasing figurative context (Colombo, 1993; Cacciari et al., 2007; Cacciari and Corradini, 2015) have been shown to affect access to figurative meaning. In fact, Giora (1997, 2002) suggests that saliency—conventionality, frequency, familiarity, contextually supported—rather than an individual factor is most relevant in idiomatic processing. Instead of differentiating between literal and non-literal processing, she separates salient processing from non-salient processing: Saliency facilitates processing as salient meanings are retrieved immediately and directly, while non-salient meanings are retrieved through default language processing and integration processes. Thus, access to figurative meaning is dependent on saliency rather than individual idiomatic properties. In contrast, Gibbs and Nayak (1989) adapted a theory of comprehension via meaning mappings after finding that decomposability, or the extent to which the meaning of individual word components in idioms contributes to the overall figurative meaning, also affects this access. In a timed response task in which participants decided whether a phrase was meaningful or not, participants were faster when responding to decomposable idioms than non-decomposable idioms, suggesting that composition via meaning mappings helps comprehension in decomposable idioms, while the missing possibility to do so in non-decomposable idioms slows down comprehension. Cacciari and Tabossi (1988) tested the effect of the presence of an idiomatic key—an aspect of predictability (see Titone and Connine, 1994b)—or a point at which the configuration of words is recognized as an idiom and found that this aspect of predictability also impacts the availability of figurative meaning (e.g., in the idiom *give the cold shoulder*, the word cold is the idiomatic key as listeners can recognize the configuration as an idiom at this point in the idiom). Presented with target words related to the idiomatic meaning and the literal meaning of an idiomatic phrase, participants' reaction times for figurative targets elicited faster responses only in highly predictable idioms while literal targets elicited faster responses only in not highly predictable idioms. Thus, the speed of access to figurative meaning can differ based on the placement of the key within the word—the sooner it occurs, the faster idiomatic meaning is available—after which point the configuration is recognized as an idiom and meaning is accessed through retrieval rather than composition. In a more recent study, Titone and Libben (2014) took several of these idiomatic differences into consideration by examining the retrieval of idioms and their figurative meaning. In their cross-modal priming study, participants made a lexical decision on target words related to the figurative meaning of the idiom in one of four positions (in two experiments). Idioms differed in familiarity, decomposability, literality, and final word predictability. They found that differing idiomatic properties modulate meaning over time in idiomatic processing, namely that high literality can hinder idiomatic processing before phrase offset; familiarity can facilitate processing at the phrase offset, and high decomposability can hinder processing 1000 ms after phrase offset. Titone and Libben interpret these results in support of a hybrid model of idiom processing in which all available information is used to facilitate processing; the result is both direct retrieval of figurative meaning and composition.

While psycholinguistic evidence generally supports processing models that take into account the heterogeneity of idioms and contexts, there are still open questions concerning their relative importance concerning access to figurative meaning in comparison to literal constituent meaning. However, research has consistently shown that not only is access to figurative meaning available online, but in some circumstances it can have an advantage over literal constituent meaning. Additionally, idiomatic processing has been consistently found to be faster than processing of comparative novel phrases (Tabossi et al., 2009). Though it's unclear whether idiom processing is essentially different from literal language processing, native speakers seem to have *mastered the art* of idiom processing.

Though there is considerably less headway in L2 idiom processing, like L1 research, L2 research addresses both the comparison of access to figurative and literal meaning and the comparison of idiomatic processing to novel language have been in focus. However, there is more variation in the results exploring overall access to figurative meaning. Using eye-tracking methods, researchers such as Conklin and Schmitt (2008), Underwood et al. (2004), and Siyanova-Chanturia et al. (2011) investigated access to figurative meaning in comparison to novel phrases. Conklin and Schmitt (2008) found that L1 and advanced L2 English users read idioms, belonging to formulaic language or multi-word expressions, more quickly than comparable novel phrases whether used figuratively or literally. Underwood et al. (2004) only partially confirmed this advantage. In a reading task in which idioms were embedded in extended contexts, both L1 and L2 English users fixated less on terminal words in idiomatic phrases than in comparable novel phrases; however, they did not find the same results for total fixation length as L1 English users did not need to look as long at final words in idiomatic phrases compared to novel phrases and L2 English users showed no significant difference in the in the length of the gaze. These results suggest a more complex picture of the processing of idioms and other formulaic language that might be heavily influenced by disadvantages particular to L2 language use. Neither of these studies, though, are able to draw conclusions concerning access to literal in comparison to figurative meaning. In contrast, in a study run by Siyanova-Chanturia et al. (2011), L1 and L2 English users read idioms in a biasing story context used figuratively (at the end of the day—eventually) literally (at the end of the day—in the evening) in comparison to a matched novel phrase (at the end of the war) and found that L1 users read idiomatic phrases, both literal and figurative uses, more quickly than novel phrases while proficient L2 users did not show differences. They did find, however, that the figurative meanings of idioms required more time to retrieve than the literal interpretation in L2 users only.

Further evidence for the priority of literal meaning in L2 idiom processing is presented by Cieślicka (2006). Following ideas about L1 language processing presented by Giora (1997), Cieślicka proposed that literal meanings are always most salient for L2 users, regardless of their frequency and familiarity, as literal words and phrases are more likely to be used and encountered by language learners. Additionally, Cieślicka examined whether the saliency of literal meaning differs with varying degrees of literality, the degree to which the idiom can be interpreted literally. In a cross-modal priming study comparing reaction times to visual targets related to literal constituents of idioms (bury the hatchet—AXE) and targets related to the figurative interpretation of the idiom (bury the hatchet—FORGIVE) in advanced Polish learners of English, Cieślicka found more facilitatory priming for literally-related targets than for figuratively-related targets in both idioms with a highly literal interpretation and those without a very literal interpretation. Cieślicka uses this as support for her *Literal Salience Model*, which suggests a priority for literal meaning based solely on L2 users' superior experience with literal meanings—saliency—compared to L2 users' limited experience with figurative meaning in their L2. Thus, unlike previous findings in L1 research, literal meaning seems to have a processing priority over figurative meaning, even in known idioms.

Among consistent research indicating that L2 language users face a variety of linguistic challenges when encountering figurative language, research in L2 idiom processing has also begun to question the effects the L1 can have on L2 figurative processing, particularly access to meaning via translation and cross-language facilitation. Research on cross-language facilitation for single word translation equivalents in bilinguals has consistently shown priming effects (Chen and Ng, 1989; de Groot and Nas, 1991; Duyck and Brysbaert, 2004; Sunderman and Kroll, 2006; Carrol and Conklin, 2014). Chen and Ng (1989), for example, found semantic facilitation using translation equivalent words as well as pictures in Chinese-English bilinguals. In line with these results, Kroll et al. (2010) point out that translation is conceptually mediated, and advanced L2 learners need not translate words, but rather access concepts immediately; thus, any facilitation might be available via a conceptual language non-selective level. Although, Brysbaert and Duyck (2010) warn that this mediation may be more difficult in the case of abstract words (see also de Groot, 1992), a potential problem for figurative language. Multi-word units, for example collocations, have also shown similar affects to single words. Wolter and Gyllstad (2011) found facilitation in English word pairs forming word-for-word translations of word pairs in Swedish compared to English-only word pairs in bilinguals of Swedish and English. Thus, it seems that a conceptual level might also mediate some multi-word units. However, there is considerably less psycholinguistic research for cross-linguistic effects in idioms. In a lexical decision task run by Carrol and Conklin (2014), highly proficient Chinese speakers of English responded faster to targets completing the final word of transliterated (word-for-word translations) Chinese idioms (draw a snake and add—FEET) than to matched controls (put it in your—DISH) and English idioms (on the edge of your—SEAT) just as L1 English users responded quickly to targets completing English idioms compared to matched controls and the Chinese idioms. Since the transliterated Chinese idioms do not have English equivalents, Carrol and Conklin suggest that access to the Chinese idiom can occur, as in their study, via a lexical route, though, their proposed dual-route also allows for access via a conceptual level of idiomatic meaning when idioms are equivalent in both languages. Their conclusion is also supported by models of late bilingual comprehension suggesting language non-selective conceptions that connect to both the L1 and L2 (see e.g., Kroll and Stewart, 1994; Kroll et al., 2010). However, as Carrol and Conklin did not find effects for L2 idioms, it remains to be seen as to whether familiar L2 idioms might also show facilitation effects comparable to those found for single words and collocations.

Research focusing on access to meaning in idioms with variable translatability from the L1 to the L2 also suggests facilitation in comprehension, production, and processing. Irujo (1986) examined the production and comprehension of English idioms of differing levels of translatability from Spanish to English in advanced Venezuelan learners of English in an offline study including a written task with multiple choice questions for recognition, an open-ended definition-writing task for comprehension, a discourse-completion task for recall, and a translation task for production. Identical idioms (one-to-one, word-for-word translations) were the easiest to comprehend and produce while different idioms (equivalent concepts not available via word-for-word translation) were the most difficult; negative interference in the form of transfer occurred in the production of partially-matching idioms (equivalent concepts and partially-matching translation). Irujo concluded that both production and comprehension can be aided in an L2 by using L1 knowledge. In a timed production task from Liontas (2002) in 3rd-year learners of Spanish, French, and German, translatability was found to be a predicting factor for speed and accuracy of production with and without context. Additionally, Liontas also found that translation is one of the most common strategies used by L2 users in comprehending idioms based on learners' written reflections. Liontas (2002, 2015) uses his findings to propose a two-stage comprehension model: prediction, eased by idioms which are the same in a learner's L1 and L2, followed by confirmation or replacement and/or reconstruction. Thus, the figurative meaning of matching idioms should be easier than non-matching or partially-matching idioms, and possible even allow for faster availability of figurative meaning. In a study comparing of bilingual idiom processing, Titone et al. (2015) looked at translatability (in this study called cross-language overlap) in French-English bilinguals—some with English L1 and some with French L1—by asking participants to decide whether sentences containing idioms were meaningful or not in a word-by-word reading task. The idioms also had differing levels of translatability, and, in some conditions, the final word was presented in French rather than English. An increase in translatability facilitated a decrease in response times for French final word idioms, but not for English final word idioms. Additionally, accuracy increased overall as translatability increased. Their analyses also included interactions for other idiomatic properties such as familiarity, predictability, and decomposability, and Titone et al. (2015) took these results as support for a hybrid model of processing, like monolingual idiom processing, in which listeners use all available information to facilitate processing.

The present cross-modal priming studies aim to look more closely at access to figurative meaning in comparison to literal meaning as well as the influence of the L1 on L2 idiom comprehension. It does seems that L2 users, like L1 users, have access to figurative meaning, though the constraints seem to be more particular than in the L1, and the priority of literal language over figurative language in the L2 is well-established. Translatability also appears to make a difference in comprehension and production of L2 idioms, and cross-linguistic effects on translations of idioms have been found, at least on a lexical level. While both of these aspects have been examined separately, they have not yet been considered together in an online study.

This cross-modal priming study includes two experiments designed to test the online availability of literal and figurative meaning in L1 and L2 listeners. In Experiment 1, native German listeners who were highly proficient in English were presented with English idioms as auditory primes. Listeners heard idioms placed at the end of short, non-biasing sentences before they decided whether a visually presented target word was an existing word of English or not. Target words were either related to the literal meaning of a constituent word of the idiom or to the figurative meaning, and both were compared to unrelated control targets. Half of the idioms had matching translations from English to German (called lexical level idioms) and half had non-matching translations (called post-lexical level idioms). Based on the L2 research presented above, we expected literally-related targets to have faster RTs than their unrelated controls. If L2 listeners also have online access to figurative meaning, then we should find facilitatory priming, that is, faster reaction times to figuratively-related targets compared to matched controls. However, based on Cieślicka's (2006) findings, it is also conceivable for the priming effect to be greater for literal targets than figurative ones. Additionally, if the offline findings of Liontas (2002) in addition to the online findings of Titone et al. (2015) hold true for this type of task, we expected the priming effect to be greater in lexical level idioms than in post-lexical level idioms. In Experiment 2, monolingual native listeners of American English were presented with the same English stimuli as in Experiment 1. Based on consistent evidence that L1 listeners have online access to figurative meaning in idioms, and evidence that literal constituents also undergo processing, we expected to find facilitatory priming for both literally- and figuratively-related targets. Additionally, we predicted that translatability should not make a difference for our monolingual English participants, since they did not know any German.

## Experiment 1

### Method

#### Participants

Sixty-five native speakers of German (48 female and 17 male; average age 24.5, range from 18 to 42) were paid a small fee to participate in the experiment. Participants had learned English later in life in instructional settings and most were students of English at the University of Tübingen at the time of testing. All participants identified themselves as skilled speakers of English. Participants reported at least 5 years of English instruction in school and or university and averaged 4.9 on a 7-point scale (1 corresponds to very poor, 7 to native-like) in their self-proficiency ratings. Seven of the participants were left-handed. None reported any hearing or visual impairments.

#### Materials

Sixty-four English idioms were selected from the English-German Database of Idiom Norms (DIN; Beck and Weber, 2016). The DIN database includes 300 English idioms and their English L1 and German L2 ratings and encoding on a variety of features. Selected idioms shared a VP syntactic structure, always beginning with a verb and ending with a noun (such as in *kick the bucket*) and were chosen based on ratings for additional attributes shown to affect processing (see e.g., Titone and Connine, 1994a,b; Titone and Libben, 2014) such as familiarity—both frequency of encounter and familiarity with the meaning—decomposability, literality—which can be defined as an idiom's potential for a literal interpretation—and word-component frequency—based on averages of individual words taken from SUBTLEXus (Brysbaert and New, 2009). Target idioms had high ratings of familiarity for German L2 raters (on a scale of 1–7, an average of 5.2 for rate of encounter and an average of 5.7 for familiarity with the meaning) in order to assure that German L2 participants in the present study would be familiar with the idioms presented. Additionally, idioms were selected with a medium literality rating (an average of 4.3) so as not to bias the experiment with highly literal idioms nor with idioms without possible literal interpretations.

Idioms varied systematically in translatability based on two of the levels of translatability laid out by Irujo (1986), Liontas (2002), and Titone et al. (2015). Irujo and Liontas used a three-level scale, and Titone et al. rightfully added to the scalarity of translatability using five levels, also including a non-language overlap condition in which there is neither an overlap in word-for-word translation nor a corresponding idiom with the same meaning in addition to adding multiple levels with partial overlap. The 32 English idioms in this study were directly translatable in that matching idioms and figurative meaning results from a word-for-word translation of the idiom from English to German (lexical level idioms) and 32 idioms were not directly translatable in that a word-for-word translation from English to German does not produce a matching idiom with equivalent meaning (post-lexical level). The post-lexical level idioms selected do, however, have idioms with equivalent meanings in German, and all idioms should be familiar based on the ratings discussed above (see Table 1 for examples). Both levels of translatability selected could be matched closely on the measures listed above, although slight differences were present in the lists. Between lists, idioms did not significantly differ in frequency of encounter, literality, decomposability, or word-component frequency, but there was a small but significant difference in familiarity with the meaning (means 6.0, 5.47; student's *t*-test, *t* = 3.991 *df* = 62, *p* < 0.001) and a significant difference in idiom word length (means 3.781, 3.156; Welch's *t*-test, *df* = 43.756, *p* < 0.05), with translatable idioms being more familiar and longer. See Table 2 for additional descriptive data of idiom norms. Idioms which only occur in the L2 and not in the L1, were not included in spite of providing a starker contrast in translatability as they could not be well-matched based on the selection criterion laid out above. However, Irujo and Liontas' results still predict that these levels will show differences in processing. All idioms were placed at the end of sentences with non-biasing contexts with as little additional information as possible, as in the sentence *John let the cat out of the bag*. Each sentence with an idiom prime was followed by one of four target words distributed across four experimental lists.

**Table 1:** **Levels of translatability**.

**English idiom**	**Type**	**Equivalent German idiom**	**Figurative meaning**
To lend (someone) an ear	LL	*Jemandem sein Ohr leihen* someone his ear lend	To listen or pay attention to someone
To kick the bucket	PL	*Den Löffel abgeben* the spoon give away	To die

**Table 2:** **Idiom norms distribution**.

	**LL**				**PL**			
	**Min**	**Max**	**Mean**	***SD***	**Min**	**Max**	**Mean**	***SD***
L2 Meaningfulness	5	6.9	6	0.56	4.25	6.3	5.47	0.5
L2 Familiarity	4.05	6.95	5.31	0.8	4.2	6.05	5	0.52
L2 Literality	2.6	5.48	4.25	0.9	2.6	5.55	4.33	0.77
Decomposable (% yes)	0.15	0.9	0.54	0.22	0.06	0.95	0.53	0.25
Constituent frequency	1.44	3.84	3.42	0.44	3.04	3.92	3.54	0.22
Idiom length (words)	2	7	3.78	1.24	3	6	3.16	0.57

LL, lexical-level idioms; PL, post-lexical level idioms.

The four target words for each idiom were literally- or figuratively-related words and their respective unrelated control words. Target words semantically related to the literal meaning were based on the last content word of the idiom and chosen from the Nelson et al. (1998) association norms database. For example, for the idiom *to pull my leg* (primed in the sentence *John likes to pull my leg.*), the target word *WALK* was chosen. The unrelated control word was matched for orthographic complexity and length (unrelated *MILK* compared to literally-related *WALK*). Targets related to the figurative meaning of the idiom were chosen based on relation to the overall meaning of the idiom. For the same idiom, the target *JOKE* was chosen, as *to pull my leg* has the meaning “to make fun of someone in a friendly way.” Similarly, figurative control targets were also controlled for orthographic complexity and length (unrelated SHIP compared to figuratively-related JOKE). Target words were also controlled for average lexical frequency. As there are no existing databases for association of figurative meaning, targets were selected by these authors (see Appendix for a list of idioms and targets used). In addition, an online ratings study using OnExp (Onea, 2011) was conducted in which 26 native speakers of American English rated the level to which the chosen figurative target reflects the meaning of the idiom in a 15-min survey. The 64 target idioms were paired with their figuratively-related targets from Experiment 1, and 20 additional filler idioms were added with targets as to elicit the full range of possible responses from very related, somewhat related, to very unrelated. Participants rated the relatedness of a target to the figurative meaning of an idiom using a 5-point scale ranging from *not at all related* (1) to *highly related* (5). Participants were not given the meaning of the idiom, and were therefore also provided with an option to select *I don't know the meaning of this phrase* rather than giving a rating. Overall, figuratively-related targets received high association ratings: post-lexical idioms received an average rating of 3.87 and lexical level idioms 4.01. In a two-sampled *t*-test, there was no significant difference in association ratings [*t*_(62)_ = 0.73, *p* > 0.46].

In addition to the 64 sentences with target idioms, an additional set of 104 filler sentences were selected. Twenty filler trials contained additional idioms, and 84 trials consisted of non-figurative sentences, meaning one-half of the trials in the experiment contained idioms. The 20 sentences with filler idioms were followed by non-words in order to ensure that a sentence containing an idiom was not always followed by a word. The remaining 84 filler trials consisted of varying sentence structures so as to provide a variety of sentence types. Filler sentences were followed visually by word or non-word targets; half of the word-targets were related and half were unrelated. In total, word and non-word targets were equally present in the trials, and all English non-words were also non-words in German. Each of the total of 168 trials (64 experimental trials and 104 filler trials) consisted of an auditory sentence prime followed by a visual target. Thirty-two of the experimental trials contained a post-lexical level idiom and 32 contained a lexical level English idiom. The experiment was presented to participants in one of four randomized lists. Filler items remained the same while lists were counterbalanced for experimental target type.

The experiment was performed using Presentation® software (Version 17.2, www.neurobs.com). Experimental sentences were recorded by a female speaker of American English (first author) in an experimental lab setting. All auditory sentences were the same for each participant, however, four counterbalanced lists were randomly allocated to participants so that conditions were evenly distributed across lists. The four lists allowed targets to be present once for each condition. Each list began with four practice trials followed in a randomized order by 64 experimental and 104 filler trials, and participants had the option of a short self-directed concentration break after trial 84. Each list was presented to an equal number of participants.

#### Procedure

Participants were tested individually in a quiet room. First, participants were given instructions in English on the lexical decision using Google Forms. Participants saw written instructions and had to answer a short series of questions to ensure uniform instructions and understanding. This was followed by a short verbal confirmation of the instructions. Participants were instructed that they would hear sentences directly followed by the appearance of an English word or non-word on the computer screen in front of them. They were instructed to listen to sentences and then decide whether the string of letters on the screen was a word of English or not. Participants were asked to make their decisions by pressing a green button with their dominant hand for “YES” and a red button with the non-dominant hand for “NO” as quickly and accurately as possible. They were also told that it was important both to listen and to respond to the visual targets, as they would be asked about what they heard after the lexical decision task. This instruction was included to ensure their continued attention throughout the experiment.

Once participants understood the instructions and answered the confirmation questions correctly, they participated in the priming study. The participants listened over closed headphones and the visual targets were presented on a laptop screen as capital white letters on a black background in size 20 font. The targets appeared on the screen right after the offset of the auditorily presented prime sentences. Reaction times were measured from the onset of the presentation of the visual target words. Participants had 2500 ms to respond, and 1000 ms after a key response, the following trial began.

The experiment concluded with a short comprehension test and a language background questionnaire. As participants were told that they would be asked about the auditory sentences following the experimental trials, participants took a short yes/no comprehension test following the experiment (see Cieślicka, 2006). The test consisted of a list of 60 sentences, of which 30 were heard in the experiment. Participants had to check “YES” or “NO” to the question of whether they heard the sentences in the experiments (participants averaged 73% correct). The entire experiment lasted about 25 min.

#### Results

Only trials with correct responses to target words were analyzed. L2 participants answered an average of 93% of targets correctly. Such a high level of performance attests the high level of L2 proficiency of our participants. Thirty-six responses with RTs longer than 1800 ms or shorter than 250 ms were considered outliers and were removed from these analyses (0.9% of the total data). Additionally, seven targets (CELERY, CHIME, EEL, NITPICK, SEAM, SHRUB, TALKATIVE) were answered correctly in 50% or less of the total trials and were excluded from the results (44 responses or 1.1% of the total data).

**Figure 1:**
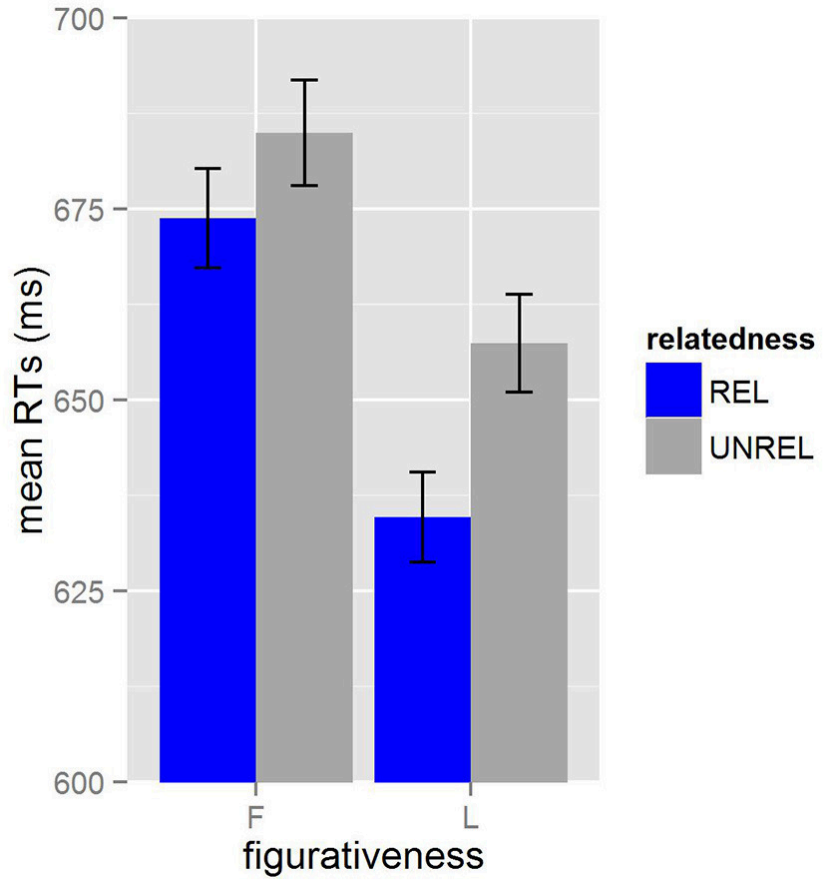
**Mean RTs (in ms) for German L2 listeners in Experiment 1**. *Figurativeness* is represented by F (figurative) and L (literal) and *relatedness* is represented by REL (related) and UNREL (unrelated).

We used R (R Core Team, 2015) and lme4 (Bates et al., 2015) to perform a linear mixed effects analysis of the relationships between *figurativeness, relatedness*, and *lexicality* on reaction times. Table 3 reports the mean RTs and inverse RTs (see Baayen, 2008) measured from target onset for each condition. The mean RTs across *figurativeness* and *relatedness* are shown in Figure 1 with error bars representing the standard error. After excluding residual data points in the inverse RTs, a total of 3.3% of the data were not included in the final analysis.

**Table 3:** **L2 mean reaction times (in ms)**.

	**Lexical**				**Post-lexical**			
	**Figurative**		**Literal**		**Figurative**		**Literal**	
	**RT**	**inverse RT**	**RT**	**inverse RT**	**RT**	**inverse RT**	**RT**	**inverse RT**
Related	686	−1.567	644	−1.669	667	−1.614	628	−1.700
Unrelated	682	−1.586	666	−1.620	695	−1.556	661	−1.633

A set of LMER models were built with inverse RTs as the dependent measure and fixed factors, centered around 0, were coded and included as follows: *figurativeness* (literal: −0.5 or figurative: 0.5), *relatedness* (related: 0.5 or unrelated: −0.5), and *lexicality* (lexical level: -0.5 or post-lexical level: 0.5). Items and participants were included as random factors, and the maximal structure suggested by Barr et al. (2013), was not included in the final model as model comparisons of individual slope adjustments—due to convergence issues with a fully maximal structure—suggested that this approach is unwarranted for this data set (see e.g., Baayen et al. submitted). *P*-values were calculated using likelihood ratio tests of the full model with the effect in question against a model excluding the effect in question in order to achieve the most parsimonious model using a backward, stepwise selection procedure. The values of the first full model can be found in Table 4.

**Table 4:** **L2 full model output**.

**Fixed effects and controls**	**Effect size**	***SE***	***t*-Value**	**Pr(>|t|)**
Intercept	−1.605	0.031	−51.03	<2e-16^***^
Figurativeness (coded)	0.072	0.022	−3.428	<0.001^***^
Relatedness (coded)	−0.044	0.022	2.013	0.045^*^
Lexicality (coded)	−0.015	0.022	−2.280	0.500
Figurativeness^*^Relatedness (coded)	0.048	0.042	1.139	0.256
Figurativeness^*^Lexicality (coded)	0.019	0.042	0.457	0.648
Relatedness^*^Lexicality (coded)	−0.048	0.043	−1.106	0.270
Figurativeness^*^Relatedness^*^Lexicality (coded)	−0.06983	0.08494	−0.822	0.411824
**Random effects**	**Variance**	***SD***		
Target	0.02401	0.1549		
Subject	0.05667	0.238		
Residual	0.07528	0.2744		

* *p < 0.05*,

*** *p < 0.001*.

After stepwise selection, the final model included *figurativeness* and *relatedness* as fixed factors (*lexicality* and all interactions were excluded because they were not significant: all *t* < 1.5, *p* > 0.10) and also items and participants as random effects. The effect of *relatedness* (*b*_*coded*_ = −0.044, *p* < 0.05) shows that related targets were significantly faster than unrelated targets [χ${}_{\left(1\right)}^{2}=$ 4.02, *p* < 0.05]. The effect of *figurativeness* (*b*_*coded*_ = 0.0717, *p* < 0.001) demonstrated that figurative targets were significantly slower than literal targets [χ${}_{\left(1\right)}^{2}=$ 11.03, *p* < 0.001].

### Discussion

This analysis shows that facilitatory priming was observed for both figuratively- and literally-related targets for L2 listeners. Even in the absence of biasing context, listeners responded more quickly to related than unrelated targets. These results are consistent with those found by Cieślicka (2006). However, the effect of *figurativeness* applies across figuratively-related and -unrelated targets, suggesting that the figurative targets were more difficult overall for L2 listeners, and a comparison of solely the figuratively- and literally-related targets should take this difference into consideration. These results suggest that non-native listeners have online access to the figurative meaning of familiar L2 idioms in addition to access to the meaning of individual component words.

We found, however, no effects or interactions involving *lexicalilty*, suggesting that the translatability of the idioms from the L1 to the L2 did not have a direct impact on processing as the model and research presented by Liontas (2002, 2015) would have predicted. Thus, while translatability may well affect comprehension strategies, there is no evidence from this analysis that suggests an extension into online processing. However, looking descriptively at the priming effects, one can see that priming effects for LL idioms are quite small in comparison to the PL idioms. The observed differences in the idiom norms do not account for this perceived difference. The difference in familiarity should predict an ease in processing for lexical-level idioms, and therefore shorter reaction times (see e.g., Titone and Libben, 2014), the opposite result from our observation. The difference in idiom length might predict more processing effort for post-lexical level idioms, supporting the results seen descriptively. However, upon closer inspection of the data, post-lexical level idioms include only six idioms that have key words occurring before the final word in the idiom, while lexical-level idioms include 10 (Beck and Weber, 2016). Again, this difference in predictability, if significant for processing, should give a processing advantage to the post-lexical level idioms rather than the lexical-level idioms. Thus, though insignificant, this perceived difference in priming cannot be explained by the typical idiomatic factors associated with priming differences.

As these results are consistent with the results of Cieślicka (2006) and inconsistent with predictions from Irujo (1986), Liontas (2002), and Titone et al. (2015), it is important to make comparisons with L1 listeners. Cieślicka's study used similar results to support evidence that L2 listeners rely more heavily on literal meaning than L1 listeners; however, her study lacked an L1 comparison. In order to make conclusions about whether these results apply only to L2 listeners, we conducted a second experiment using the same materials and methods on L1 listeners.

## Experiment 2

### Method

The method was the same as in Experiment 1.

#### Participants

Forty native speakers of American English were paid a small fee to participate in the experiment. All participants grew up in English-speaking households and did not speak or know any German. Participants were students at the University of Maryland in College Park, MD, USA. L1 participants ranged from 19 to 31 years old with an average of 22.5 years old. Five of these participants were left-handed. There were 30 female and 10 male L1 participants. Participants reported no hearing or visual impairments.

#### Materials

The materials were the same as in Experiment 1.

#### Procedure

The procedure was the same as in Experiment 1, except that the experiment was conducted in the laboratory at the University of Maryland Language Science Center.

#### Results

The analyses include again only correct responses to targets. L1 participants correctly answered an average of 97% of targets. Eleven responses with RTs longer than 1800 ms or shorter than 250 ms were considered outliers and were removed from these analyses (0.004% of the total data). The data from one participant was also excluded because she did not follow the instructions. The comparable averages for correct responses in Experiments 1 (93%) and 2 (97%) further confirm the high level of proficiency of our L2 participants in Experiment 1.

**Figure 2:**
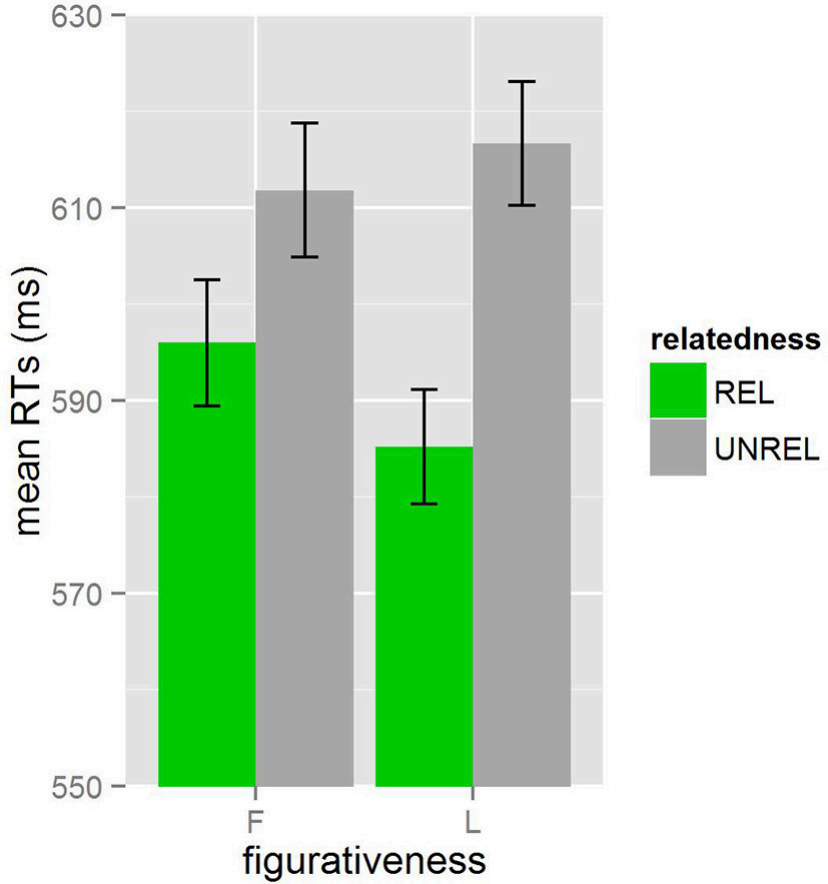
**Mean RTs (in ms) for English L1 listeners in Experiment 2**. *Figurativeness* is represented by F (figurative) and L (literal) and *relatedness* is represented by REL (related) and UNREL (unrelated).

As in Experiment 1, we used R (R Core Team, 2015) and lme4 (Bates et al., 2015) to perform a linear mixed effects analysis of the relationships between *figurativeness, relatedness*, and *lexicality* on reaction times. Table 5 reports the mean RTs and inverse RTs measured from target onset for each condition. Figure 2 shows mean RTs for *figurativeness* and *relatedness*, with error bars representing standard error. A total of 3.7% of the data were not included in the final analysis after exclusion of any remaining residuals.

**Table 5:** **L1 mean reaction times (in ms)**.

	**Lexical**				**Post-lexical**			
	**Figurative**		**Literal**		**Figurative**		**Literal**	
	**RT**	**inverse RT**	**RT**	**inverse RT**	**RT**	**inverse RT**	**RT**	**inverse RT**
Related	601	−1.791	587	−1.831	595	−1.830	584	−1.856
Unrelated	603	−1.788	631	−1.744	622	−1.732	606	−1.791

Using inverse RTs as the dependent measure and the fixed factors, coded and centered around 0, of *figurativeness* (literal: −0.5 or figurative: 0.5), *relatedness* (related: 0.5 or unrelated: −0.5), and *lexicality* (lexical level: −0.5 or post-lexical level: 0.5) as well as items and participants as random factors (see e.g., Barr et al., 2013; Baayen et al. submitted), a set of LMER models were constructed. *P*-values were calculated using likelihood ratio tests of the full model with the effect in question against a model excluding the effect in question in order to achieve the most parsimonious model using a backward, stepwise selection procedure. Table 6 displays the full output from our original model.

**Table 6:** **L1 full model output**.

**Fixed effects and controls**	**Effect size**	***SE***	***t*-Value**	**Pr(>|t|)**
Intercept	−1.793	0.052	−34.361	<2e-16^***^
Figurativeness (coded)	0.023	0.019	1.183	0.238
Relatedness (coded)	−0.060	0.020	−3.09	0.002^**^
Lexicality (coded)	−0.014	0.020	−0.721	0.472
Figurativeness^*^Relatedness (coded)	0.034	0.039	0.887	0.376
Figurativeness^*^Lexicality (coded)	0.046	0.039	1.189	0.236
Relatedness^*^Lexicality (coded)	−0.040	0.039	−1.013	0.312
Figurativeness^*^Relatedness^*^Lexicality (coded)	−0.130	0.078	−1.669	0.096
**Random effects**	**Variance**	***SD***		
Target	0.014	0.119		
Subject	0.102	0.32		
Residual	0.096	0.3103		

^*^p < 0.05,

** *p < 0.01*,

*** *p < 0.001*.

Only *relatedness* was included in the final model as a fixed factor (all interactions, *figurativeness*, and *lexicality* were excluded because they were not significant: all *t* < 2, *p* >0.05) in addition to items and participants as random factors. The three-way interaction approaches, but does not reach significance. The effect of *relatedness* (*b*_*coded*_ = −0.0607, *p* < 0.01) shows that responses to related targets were significantly faster than responses to unrelated targets [$\chi_{\left(1\right)}^{2}=$ 9.1494, *p* = 0.002488].

### Discussion

As in Experiment 1, facilitatory priming was found for both figuratively- and literally-related targets in L1 listeners. Even in the absence of biasing context, listeners responded more quickly to related than unrelated targets, as expected based on previous findings (Ortony et al., 1978; Swinney and Cutler, 1979; Cacciari and Tabossi, 1988). While the priming effect, however, is numerically larger for literally-related targets than for figuratively-related targets, this difference is not significant. There are a multitude of explanations for this potential difference in priming effects. Cacciari and Tabossi's (1988) study suggest that figurative priming only increases to the level of literal priming for idioms with high levels of predictability. Our study did not account for predictability, and instead relied on controlling factors such as familiarity and syntactic structure, as familiarity and predictability correlate (Titone and Connine, 1994a) and syntactic structure can also be an indication of predictability (i.e., idioms beginning with common verbs such as “take the…” and “get the…” will be similarly unpredictable before the final word). Colombo (1993) had similar findings in a cross-modal priming study and suggested that the difference in nature of the semantic relationship of figuratively- and literally-related targets to the idioms might explain the differences. While our literal targets are single word associations, our figurative targets are semantic relations of the idiomatic phrase to a related word. Colombo and Williams (1990) suggest that priming effects are less stable in semantic relations in comparison to associative relations (as cited in Colombo, 1993).

The results differed from those in Experiment 1 as there was no effect for *figurativeness* in the L1. Though the visual data in Figure 2 displays figurative targets as generally slower than literal targets also for L1 participants, this effect did not reach significance in Experiment 2. This suggests that the effect found in L2 listeners is likely a consequence of the difficulty of the figurative words. While the target lists were controlled for frequency based on the COBUILD frequencies per million and syllable structure provided in Max Planck Institute for Psycholinguistics (2001), this process cannot exclusively control for L2 difficulty. Furthermore, we did not place restrictions on word class or concreteness of the term, as it was most important that our subjects identify the figuratively-related target with the meaning of the idiom, and idiomatic meaning is often expressed most closely with abstract words rather than the more concrete words from the literal word associations (e.g., BEHAVE vs. FOOTBALL). It is possible that the differing word classes or levels of concreteness of the word might also have impacted difficulty; while L1 listeners were less challenged by this variation, L2 listeners appear to have reacted differently. As Brysbaert and Duyck (2010) pointed out, L2 translation studies consistently find that translation of abstract words differ from concrete words (see also Peterson et al., 2001). Thus, the slower responses to figuratively-related targets compared to figuratively-related targets might be an expression of an effect of concreteness.

Another difference that can be observed between the experiments is that the L1 listeners in Experiment 2 had overall faster reaction times than the L2 listeners in Experiment 1. This reflects common findings that suggest that L2 comprehension is more difficult and typically slower (e.g., see review in Cutler, 2012) and does not necessarily reflect a low level of proficiency of the L2 participants. Rather, the similarity in L1 and L2 behavior displayed in the priming effects for relatedness confirms a high level of proficiency of the L2 participants. Both L1 and L2 listeners displayed access to figurative meaning in addition to literal meaning, though priming effects are somewhat smaller for L2 participants.

Although the lack of significance in the effect of *lexicality* implies that L1 listeners were also not impacted by the translatability of the idioms from English to German, an observation of the data from both groups still suggests that the priming effect is stronger for post-lexical idioms than lexical level idioms. As our descriptive data of idiom norms did not provide an explanation for this observation an additional data analysis is included. For this reason, in addition to the observation that our three-way interaction in the L1 model approaches significance, we expanded our models in another analysis of the data to include the available idiomatic norms as fixed effects. In addition to the fixed factors, coded and centered around 0, of *figurativeness* (literal: −0.5 or figurative: 0.5), *relatedness* (related: 0.5 or unrelated: −0.5), and *lexicality* (lexical level: −0.5 or post-lexical level: 0.5) on inverse reaction times, we added individual effects for *L2 meaningfulness, L2 familiarity, decomposability* (based on percent positively rated decomposable), *L2 literality, idiom constituent frequency*, and *idiom length*. In addition to the three-way interactions between *figurativeness, relatedness*, and *lexicality* and the individual two-way interactions, interactions between lexicality and each of the idiomatic norm measures were also included in L1 and L2 models. Items and participants as random factors were also included. Our results remained stable, and we found no significant interactions with *lexicality* and the individual idiomatic norm measures in neither the L1 nor the L2 models. However, in the L2 models, decomposability (*b* = 0.081, *p* = 0.090) approaches significance. Additionally, L2 meaningfulness as a fixed effect approaches significance (*b* = 0.064, *p* = 0.079) in the L1 model. While this suggests that for L2 listeners, decomposability might have an effect on processing, and for L1, meaningfulness could play a role. However, their lack of significance and the stability of our initial results further supports our controls across these measures. Interestingly, based on the differences in averages between the two groups (significant differences in familiarity and word-length), we would have predicted a further boost for processing in lexical-level idioms (see e.g., Titone and Libben, 2014). However, as all other effects remain consistent across both experiments and listener groups, we can conclude that there is not a fundamental difference connected with translatability that we can ascertain between the two sets of idioms that overtly affected processing.

In a final additional analyses, we combined both data sets into one analysis to confirm whether our findings remain consistent, and to confirm any differences between the groups. Our full model again used inverse RTs as the dependent measure and the fixed factors, coded and centered around 0, of *figurativeness* (literal: −0.5 or figurative: 0.5), *relatedness* (related: 0.5 or unrelated: −0.5), *lexicality* (lexical level: −0.5 or post-lexical level: 0.5), and *language* (L1: 0.5 or L2: −0.5) as well as items and participants, which included random slopes (including language across subjects), as random factors (see e.g., Barr et al., 2013; Baayen et al. submitted). *Relatedness* remains significant in the full analysis (*b* = −0.056, *p* < 0.01), confirming our initial results. An additional effect for *language* was present (*b* = −0.197, *p* < 0.01), validating our observation that L2 listeners performed significantly slower than L1 listeners. Although *figurativeness* also appears as a significant factor in this full analysis (*b* = 0.046, *p* < 0.05), a highly significant interaction between language and figurativeness (*b* = −0.050, *p* < 0.001) motivates a split of the data naturally presented in this paper in each of the two experiments. Thus, our final analysis can also confirm an overall difference in performance between our two listener groups in the experiments individually presented.

## General discussion

The primary purpose of this study was to extend the body of evidence concerning L2 idiom processing and access to figurative meaning in comparison to literal constituent meaning and to test whether this access is impacted by the L1 via translatability of idioms. While the results of Experiment 2 can be used as a control for comparison to the L2 results, the findings in Experiment 1 are also a reflection of current findings in psycholinguistic literature concerning L1 idiom processing. The availability of both literal constituent meaning in addition to the figurative meaning confirms findings by Cacciari and Tabossi (1988), Gibbs et al. (1989), Sprenger et al. (2006), and Titone and Connine (1994b), for example. When examining these results against the performance of our L2 participants in Experiment 1, there does not seem to be evidence for an L2 mode of idiom processing that significantly differs from the way idioms are processed in an L1.

In addressing access to figurative meaning in comparison with literal meaning in particular, the results of these experiments can be interpreted in light of current models of L1 and L2 idiom processing. The findings of this study generally do not support early stage models for L1 idiom processing, as they present idiom processing as semantically empty (see e.g., Cacciari, 2014). The speed and availability of the literal constituent meaning in addition to activation of the figurative meaning suggests that neither was literal composition aborted, as suggested by the early models presented by Swinney and Cutler (1979) and Gibbs (1980), nor is it likely that this meaning only became available after obligatory composition and rejection of the literal meaning, as proposed by the standard pragmatic view supported by Bobrow and Bell (1973).

Taking the effects of individual idiom properties into consideration, our data are largely compatible with several L1 and L2 processing theories, while casting doubt on others. Although theories on decomposability are somewhat compatible, the major theories considering this property still present problems. The *Idiom Decomposition Hypothesis* proposed by Gibbs and Nayak (1989) suggests that literal composition aids idiomatic processing; thus, decomposable idioms are comprehended more quickly than non-decomposable idioms. While we cannot make any claims about advantages of one or the other (and this tenant of the hypothesis has been criticized by conflicting evidence—see e.g., Cutting and Bock, 1997; Libben and Titone, 2008) since the idioms in the present study were of an medium level of decomposability, it is clear that some level of composition is taking place. Thus, while this theory fits our data more accurately than theories that assume an either-or approach to composition and retrieval in the processing of idioms, we cannot present evidence for all aspects of this approach. Additionally, as Cieślicka (2006) points out, the role the literal meaning of the individual words play in constructing figurative meaning is somewhat vague. A similar L2 model proposed by Abel (2003) in her *Model of Dual Idiom Representation* is generally compatible with our findings; though, this model differs as it explains representation rather than processing. This model assumes that both non-decomposable and frequently encountered idioms are represented in the mental lexicon by idiom entries on the conceptual level, as seen in the *Idiom List Hypothesis*, while decomposable idioms are represented via lexical entries of the individual constituent words. Abel argues that encountering an idiom often enough allows storage to occur, though, decomposability will also facilitate a faster retrieval if no entry has yet been created. Our study included idioms that were highly familiar, not only to L1 speakers, but also to L2 speakers in the target language group, suggesting that many of the idioms should be available merely via direct retrieval—a notion already deemed problematically vague as the availability of literal meaning is also consistent even in these highly familiar idioms. What's not clear is whether literal composition continues in the case of retrieval, in which case, we would have expected to find strong figurative activation to occur in opposition to strong literal activation, a notion our data do not represent. However, if retrieval accompanies further composition, our data can generally support this model of representation.

The *Configuration Hypothesis* (Cacciari and Tabossi, 1988), suggesting that literal word processing occurs until an idiomatic key is reached, is somewhat compatible with our findings. The assumption that both literal constituent meaning as well as figurative meaning can be activated is confirmed by our findings. Cacciari's (2014) new look at this hypothesis also addressed the problem of literal constituent availability in retrieval-based models of idiom processing. Cacciari points to evidence presented by Peterson et al. (2001) that suggest that individual word meanings, though activated, might only be done so for purposes of syntactic control and not integrated into the meaning of the sentence. This view is also supported by the ERP study presented by Rommers et al. (2013) in which predictable words in idioms were replaced by semantically similar words, and while this produced N400 effects for predictable literal phrases, the effect was not present for idioms. Thus, assuming activation of literal meaning is possible in spite of retrieval in predictable idioms, the process posed by the *Configuration Hypothesis* is supported by our data. However, the difference confirmed in predictability (translatable idioms had more predictable idioms than non-translatable idioms) could not be confirmed by the data, though, the advantage for predictable idioms in comparison to non-predictable idioms was not addressed in this study.

Two alternative propositions for this simultaneous activation, also supported by our data, are presented by hybrid models such as the *Superlemma Hypothesis* (Sprenger et al., 2006) and the *Constraint-Based Model of Idiom Processing* (Titone and Connine, 1999; Libben and Titone, 2008; Titone and Libben, 2014). In Sprenger et al.'s production hypothesis, continued processing of constituent parts is not discounted after an idiom is identified. Based on psycholinguistic evidence from production tasks that constituent and phrasal processing occur simultaneously, this hypothesis suggests that activation occurs for individual word lemmas and spreads to super lemmas at the lexical-syntactic level representing the meaning of the idiom. Thus, this hybrid model sees processing as a web of meaning taking place on several levels, allowing literal constituent meaning to spread and reach figurative phrasal meaning once enough information is present, very much like the Configuration Hypothesis. However, as Cacciari (2014) points out, this hypothesis assumes that idiomatic meaning is built up beginning with the first word of an idiom rather than retrieved upon cue. As participants reacted at the offset of the idiom, our data does not support one view more than the other. The *Constraint-Based Model* answers some of these uncertainties in its account that meaning is built over time using all available information; thus, while some meaning may be available upon encountering the first word, it is likely that full activation will only occur in the presence of more available information (e.g., context). Rather, comprehension will use all available information, as it is made available, causing different information to be available at different times. Our data supports this view as literal constituent meaning seems to be available faster than figurative phrasal meaning; however, both are available online. Additionally, this model is also supported by our analyses including idiomatic norms. Though none of the idiomatic norms reached significance as fixed effects, we conclude that this is due to the similarity of our idiomatic items and predict that these norms might reach significance, as trends were already present, in the presence of more idiomatic diversity. Thus, an account of processing that also takes these norms into consideration is highly compatible with our data.

While our data replicates the findings of Cieślicka's (2006) study, we do not propose that our findings entirely support the *Literal Salience Model.* Like Giora's (1997) *Graded Salience Hypothesis*, this model suggests that saliency always has processing priority, regardless of the figurativeness or literality of a given phrase. In this case, L2 listeners should always respond faster to literal meaning, as it is generally more salient for L2 listeners. While our L2 results also produced this general effect, it is repeated in our L1 findings—something we would not expect if we assume that L1 listeners should often find the figurative meaning to be more salient (Giora, 1997, 2002). While the availability of meaning does confirm that literal constituent meaning plays a role in processing idioms, we are not prepared to assume a necessary priority. While the eye-tracking evidence from Siyanova-Chanturia et al. (2011) also supports this notion generally, it is rather frequency—an important aspect of saliency—that is important, an idea that does not preclude literal priority, particularly for advances L2 users. It should also be noted that Cieślicka's (2006) study did not include an L1 group for comparison, and thus makes the assumption that L1 processing is fundamentally different than L2 research based on different findings in studies conducted on solely L1 groups (Cacciari and Tabossi, 1988, for example). We would argue that the similarity of behaviors in Experiments 1 and 2 suggests that L2 processing generally mirrors L1 processing.

The model addressing translatability, specifically Liontas' (2002, 2015) *Idiom Diffusion Model of Second Languages*, is also not supported by our data. We expected to find a significant difference in the priming effect of post-lexical level idioms compared to lexical level idioms in Experiment 1 and not in Experiment 2. We found no evidence that processing was affected by translatability. Additionally, the similarity of L1 and L2 behavior further poses problems for this theory. One possible reason our data might not reflect this model is that our listeners were highly proficient, and Liontas (2002) focused on 3rd year learners. Just as Abel (2003) suggests that L2 idiom entries occur on a conceptual level after L2 users encounter them over time, Duyck and Brysbaert (2004) suggest that proficient L2 users directly map L2 words to conceptual meaning when there is a direct overlapping of L1 and L2 words. Therefore, the available evidence that single words are translated as we hear them (see e.g., Blumenfeld and Marian, 2007) may not affect the activation of figurative meaning in the L1 if the L2 listeners directly access meaning. We might then still expect to find an effect of translation in less-proficient L2 listeners as they may not have direct mapping from the L2 to the conceptual figurative meaning. However, it is difficult to test this as an examination of idiomatic processing requires that listeners are familiar with the figurative meaning in order to test its availability—something we would not expect of less-proficient listeners. Additionally, we did not test the full scale of translatability presented by Liontas (2015) or Titone et al. (2015), as we omitted a partial overlap present in both studies and all of our idioms had matching counterparts in both languages, unlike in the study from Titone et al. While a control group with no overlap might have increased the distance in translatability, these idioms tended to have a low L2 familiarity, an important selection criteria for our experimental idioms. We omitted intermediate levels, however, to ensure that enough distance in translatability was present to still impact processing. Therefore, while offline evidence presented by Liontas as well as Irujo (1986) makes the case for facilitation in comprehension and production and Titone et al. (2015) find an online effect in code-switched idioms, we were not able to replicate these results in online processing for proficient L2 listeners. Experimental materials and task-type may have played a role in these findings (see e.g., Libben and Titone, 2008).

## Conclusion

In summary, the results of the present study show that both L1 and L2 listeners show access to figurative meaning as well as literal constituent meaning in the absence of a clearly biasing context. Additionally, we did not find that the translatability of idioms from listeners' L1 to their L2 had a measurable impact on processing. We take these results as evidence that highly proficient L2 listeners process figurative meaning in a way that is not entirely unique from L1 listeners—namely, it is impacted by the same factors that L1 idiom processing is. While our intent was not to generalize about models of processing or representation, we can interpret our results as supporting models that maintain direct mapping of L2 words to a conceptual language non-selective level (e.g., Abel, 2003; Brysbaert and Duyck, 2010; Kroll et al., 2010; Carrol and Conklin, 2014) and include the possibility for both retrieval and composition (e.g., Titone and Connine, 1999; Libben and Titone, 2008). Thus, for highly proficient L2 users, as the results of Titone et al. (2015) suggest, idiom processing follows the same routes as L1 idiom processing. This conclusion is also in line with Matlock and Heredia (2002), who suggest that beginning learners first access the literal meaning and translate it into their L1, then try to get the literal meaning before they can access the figurative language. Over time, learners are able to bypass the first two steps. L2 listeners may be slower or have more difficulty processing some figurative language, however, as they become more proficient, we conclude that any differences in processing are likely due to general L1 and L2 differences rather than a distinct manner of processing.

## Statement of ethics

This study was carried out with the recommendations for ethical guidelines of the Collaborative Research Center 833, the University of Tübingen, Germany. We employed non-invasive methods that caused neither temporary nor lasting harm to humans. Participants offered to take part of their own free will. All participants gave prior informed consent in accordance with the Declaration of Helsinki and were allowed to withdraw from the experiments at any time.

## Author contributions

SB and AW both contributed to the conception, design, and interpretation of the results. SB was primarily responsible for drafting and revising based on the critical comments and input from AW. Both SB and AW approved the final version and can be held accountable for aspects of accuracy and integrity.

## Funding

Funding for this article was provided by the Collaborative Research Center (SFB) 833, “The Construction of Meaning—the Dynamics and Adaptivity of Linguistic Structures” and the cooperation “Language Structures in German and English” between the University of Maryland and the University of Tübingen.

### Conflict of interest statement

The authors declare that the research was conducted in the absence of any commercial or financial relationships that could be construed as a potential conflict of interest.

## Appendix

**Table A1:** **Experimental Items**.

**DIN Database Reference Number^a^**	**Translatability**	**Sentence Context and *Idiom***	**Figurative**		**Literal**	
			**Related**	**Control**	**Related**	**Control**
5	Lexical	They are always *armed to the teeth*.	Weapon	Salad	Dentist	Sentence
10	Lexical	For years she's been *beating the drum*.	Support	Laughter	Band	Song
18	Lexical	I think he was *building castles in the air*.	Daydream	Coffee	Cloud	Frog
19	Lexical	The teacher *bit that boy's head off*.	Scold	Trip	Body	Daisy
27	Lexical	John was *born with a silver spoon*.	Rich	Thick	Fork	Bark
29	Lexical	Let's *break the ice*.	Cold	Social	Towel	Slot
38	Lexical	The boy *couldn't believe his ears*.	Doubt	Sheep	Hear	Mail
52	Post-lexical	It's going to *cost an arm and a leg*.	Expensive	Attention	Foot	Soup
54	Lexical	We need to *cover the territory*.	Scope	White	Area	Radio
62	Post-lexical	John will *deliver the goods*.	Result	Cement	Product	Pretend
63	Lexical	Today I will *do the honors*.	Announce	Account	Award	Water
65	Post-lexical	It didn't *do the trick*.	Satisfy	Gravity	Magic	Feature
70	Lexical	Last night he *dropped a bombshell*.	Surprise	Lemon	Explode	Produce
74	Post-lexical	I heard about her *fall from grace*.	Disfavor	Develop	Prayer	Cracker
75	Lexical	It didn't *fall on deaf ears*.	Ignore	Hanger	Nose	Bar
89	Post-lexical	I told him to *get a grip*.	Control	Shelter	Hold	Shirt
91	Post-lexical	John *has the blues*.	Sad	Seal	Music	Nation
105	Lexical	He *gave me the cold shoulder*.	Ignore	Autumn	Arm	Beet
106	Lexical	I think he will *go the limit*.	Endure	Elbow	Boundary	Celery
108	Post-lexical	The thing just *went to pieces*.	Upset	Album	Parts	Rent
113	Post-lexical	He wanted to *have a go*.	Try	Spa	Move	Cheek
118	Lexical	He might have *had cold feet*.	Nervous	Timber	Toes	Tax
120	Lexical	He doesn't *have the heart*.	Brave	Trim	Organ	Agent
127	Post-lexical	John *hit the road*.	Leave	Rate	Highway	Shadow
132	Post-lexical	He tried to *hitch a ride*.	Lift	Raft	Horse	Yarn
145	Post-lexical	His uncle *kicked the bucket*.	Die	Zoo	Pail	Boat
147	Post-lexical	It's clear that he *knew the score*.	Aware	Apart	Points	Belts
153	Lexical	I asked her to *lend me an ear*.	Listen	Logic	Head	Game
155	Lexical	John *let the cat out of the bag*.	Reveal	Season	Box	Pin
162	Lexical	He did it without *losing face*.	Disgrace	Purpose	Eyes	Eel
163	Post-lexical	John worried he might *lose his touch*.	Unable	Animal	Finger	Meadow
164	Lexical	At some point the man *lost his thread*.	Stray	Tree	Needle	Label
170	Lexical	He does that to *make a living*.	Earn	Arch	Dead	Moan
172	Post-lexical	John tried to *make a pass*.	Flirt	Nurse	Throw	Shrub
180	Lexical	The man *missed the mark.*	Fail	Keep	Spot	Stack
200	Lexical	I heard John *played a part*.	Influence	Objective	Piece	Gaze
201	Post-lexical	They decided to *play it by ear*.	Improvise	Anything	Wax	Tab
204	Post-lexical	The woman just tried to *play the game*.	Behave	Message	Football	Novel
207	Post-lexical	He is going to *pop the question*.	Propose	Swallow	Answer	Orange
209	Lexical	Last night she *poured her heart out*.	Confide	Mandate	Lung	Bird
211	Post-lexical	John likes to *pull my leg*.	Joke	Ship	Walk	Milk
212	Lexical	He might have to *pull the plug*.	Stop	Clip	Electricity	Creativity
220	Lexical	He asked us to *read between the lines*.	Infer	Envy	Text	Silk
223	Post-lexical	He asked if it *rings a bell*.	Remind	Suspend	Chime	Thief
229	Lexical	He *rules with an iron fist*.	Reign	Sink	Knuckle	Letter
234	Lexical	He told me to *save my skin*.	Rescue	Moment	Soft	Mask
241	Post-lexical	She finally *saw the light*.	Accept	Affair	Dark	Short
248	Lexical	That's his way of *showing his flag*.	Promote	Circle	Wave	Hip
250	Post-lexical	She spent a while *sitting on the fence*.	Choice	Shave	Yard	Wind
252	Post-lexical	He accidentally *let it slip his mind*.	Forget	Shadow	Brain	Truck
255	Post-lexical	I think John *spilled the beans*.	Confess	Level	Rice	Fox
257	Lexical	Let's not *split hairs*.	Nitpick	Purchase	Brush	Speak
258	Post-lexical	Let's *split the difference*.	Compromise	Talkative	Similar	Fabulous
263	Post-lexical	It's supposed to *stir the blood*.	Provoke	Tribal	Red	Lock
275	Lexical	He decided to *take the bull by the horns*.	Confront	Background	Cow	Mop
276	Post-lexical	They sure *took the cake*.	rule	Seam	Birthday	Journey
278	Post-lexical	She will have to *take the stand*.	Witness	Curtain	Sit	Fan
281	Lexical	She was just *throwing money out the window*.	Waste	Bench	Glass	Flame
283	Post-lexical	She *tied the knot*.	Marry	Pillow	Rope	Mouse
288	Post-lexical	Last night John *turned a corner*.	Improve	Attract	Street	Own
289	Post-lexical	John is responsible for *turning the tables*.	Advance	Rhythm	Chair	Shot
296	Post-lexical	Don't *waste your breath*.	Ineffective	Unattended	Oxygen	Element
297	Lexical	I don't think he *wears the pants*.	Command	Debate	Leg	Moon
300	Post-lexical	I think he would *give the world*.	Crave	Chat	Universe	Library

a Beck and Weber, 2016.
